# Comparison between Cryopreserved and Dehydrated Human Amniotic Membrane Graft in Treating Challenging Cases with Macular Hole and Macular Hole Retinal Detachment

**DOI:** 10.1155/2020/9157518

**Published:** 2020-07-07

**Authors:** Yu-Hsuan Huang, Der-Chong Tsai, Lei-Chi Wang, Shih-Jen Chen

**Affiliations:** ^1^Department of Ophthalmology, Asia University Hospital, Taichung, Taiwan; ^2^Department of Ophthalmology, National Yang-Ming University Hospital, Yilan, Taiwan; ^3^School of Medicine, National Yang Ming University, Taipei, Taiwan; ^4^Department of Pathology and Laboratory Medicine, Taipei Veterans General Hospital, Taipei, Taiwan; ^5^Department of Ophthalmology, Taipei Veterans General Hospital, Taipei, Taiwan

## Abstract

**Purpose:**

To evaluate the surgical outcomes of cryopreserved and dehydrated human amniotic membrane (hAM) graft transplantation for macular hole (MH) and macular hole retinal detachment (MHRD) repair.

**Materials and Methods:**

This retrospective, interventional case series was conducted in two hospitals. Two types of hAM grafts, namely, the dehydrated form (AmnioGen, HCT Regenerative, Taiwan) and the cryopreserved form (AmnioGraft, Bio-Tissue, Miami, FL), were consecutively used in MH surgeries. Anatomical and functional outcomes between the 2 types of hAM grafts were compared.

**Results:**

Seventeen patients (mean age: 62.1 ± 10.0 years, 9 (52.9%) males) were enrolled. Of them, 11 patients had persistent MH, 3 had MH without prior surgery, and 3 had MHRD. A cryopreserved hAM graft was used in 10 patients, and a dehydrated hAM graft was used in 8 patients. One patient used a cryopreserved hAM in the first MH surgery and a dehydrated hAM in the second surgery for extramacular hole with retinal detachment. After a 6-month follow-up, 13 (76.5%) patients had sealed MHs. The average visual acuity (VA) of cases with sealed MHs improved from 1.38 ± 0.62 to 1.12 ± 0.47 logMAR (*p*=0.03). In the other 4 cases with persistent MH, 3 had graft dislocation and 1 had a reopened MH with graft contraction. There were no significant differences in closure rate (80.00% vs. 71.43%, *p*=0.68) or VA improvement (0.19 ± 0.37 logMAR vs. 0.15 ± 0.41 logMAR, *p*=0.85) between the 2 kinds of hAM graft.

**Conclusion:**

This preliminary case series showed that both cryopreserved hAM and dehydrated hAM are feasible alternative grafts for either persistent or recurrent MH. Both approaches have similar anatomical and functional outcomes.

## 1. Introduction

Persistent macular holes (MHs) and macular hole retinal detachment (MHRD) or primary MHs and MHRD with large holes are very challenging to manage and often cause severe visual impairment, especially in highly myopic eyes [1–4]. The reported rates of persistence and reopening of MHs are 4.8%–9.2% in previous studies where surgeons had used different materials such as internal limiting membranes, lens capsules, and autologous retinal free-flaps to fill the hole [5–13].

Human amniotic membrane (hAM) grafts have been widely applied in the treatment of ocular surface disorders for a long time, as they serve as a scaffold for cell growth and migration, facilitate re-epithelialization by providing growth factors, and reduce inflammation and scarring by inhibiting TGF-*β* signal transduction [14–17]. Recently, Rizzo et al. and Caporossi et al. reported subretinal implantation of hAM grafts in the treatment of recurrent MH and retinal detachment with large macular tears and retinal breaks. Encouraging anatomical and functional outcomes were reported in these studies [18–20]. Cryopreserved hAM grafts from a tissue bank, instead of commercialized tissue products, were applied in these studies.

Two types of commercial hAM graft products are now available for medical use: cryopreserved and dehydrated amniotic membranes. They differ in morphology and structure due to differences in processing [21]. Both types of commercial hAM graft have been utilized in plastic and orthopedic surgery, such as for burns [22], diabetic foot ulcers [23, 24], tendinopathy, and arthritis [25, 26]. No significant differences between the outcomes of either type of hAM graft for these procedures were reported [23]. These commercially available hAM grafts are commonly used for treating ocular surface diseases [27], yet their differences in therapeutic efficacy remain unclear due to lack of large comparable studies. In this study, we aimed to report the surgical outcomes of applying cryopreserved or dehydrated hAM graft transplantation for persistent MH, recurrent MHRD, or primary MH and MHRD with macular holes larger than 500 *μ*m.

## 2. Materials and Methods

This retrospective, consecutive case series was conducted in Taipei Veterans General Hospital and National Yang-Ming University Hospital. This study adhered to the Declaration of Helsinki and was approved by the Institutional Review Boards of Taipei Veterans General Hospitals (2019-06-023CC) and National Yang-Ming University Hospital (2019A026). Informed consent for surgery was obtained from all the patients.

We retrospectively reviewed the medical records of patients who received vitrectomy and hAM graft transplantation from October 2018 to August 2019. All patients had MH with or without retinal detachment before surgery and were followed up for at least 6 months. All surgeries were performed by the same experienced surgeons (S. J. Chen and D. C. Tsai). Detailed ophthalmic histories were taken preoperatively, including previous ocular diagnoses, duration of MH, previous surgeries, or traumatic injury. All patients underwent a thorough ophthalmic examination before and after the surgery (2 weeks, 1 month, 3 months, and 6 months postoperatively), including best-corrective visual acuity (BCVA; converted to logMAR), axial length, slit-lamp biomicroscopy, fundus color photography, optical coherence tomography (OCT), and optical coherence tomography angiography (OCTA; AngioVue; Optovue, Fremont, CA). The size of the macular hole was defined as the minimum diameter of the hole measured on the OCT image using the caliper function.

All cases underwent standard 23-gauge 3-port microincision vitrectomy with a Constellation Vision System (Alcon Surgical, Fort Worth, TX). After completing vitrectomy and internal limiting membrane peeling for primary MH and MHRD, the hAM graft was trimmed according to the size of the MH by a 1 mm- or 1.5 mm-skin biopsy punch or simply cut using scissors with the aid of a caliper. The size of the graft was designed to be approximately 300–500 *μ*m larger than the MH size calculated according to OCT before surgery. One of either the dehydrated (AmnioGen, HCT Regenerative, Taiwan) graft or the cryopreserved hAM (AmnioGraft, Bio-Tissue, Miami, FL) graft was randomly selected for each patient according to the availability of the graft at the time of surgery. For easier identification of the two sides of the graft, the epithelial side of the graft was stained with Brilliant Blue G (at Taipei Veterans General Hospital) or indocyanine green (at National Yang-Ming University Hospital) before cutting. The trimmed hAM graft was then gently implanted into the MH with intraocular forceps and secured by trapping the graft edge beneath the retina 360 degree, if possible, with the stromal side in contact with the retinal pigmented epithelium (RPE) or at least under the retina as much as possible and left the rest of the graft above the retina in patients with large MHs and concomitant RD. To make sure that the graft was not inserted upside down, we identified the sticky stromal side with the intraocular forceps before implantation into the MH, as described by Caporossi et al. [19]. When the graft was in the correct position, fluid-gas exchange was performed, followed by 20% sulfur hexafluoride (SF_6_) or silicone oil. All patients maintained the prone position immediately after the surgery and kept the face-down position for one to two weeks. They stayed in the hospital for 3–5 days and were then discharged. We instructed the patients to maintain the prone position at home for at least 16 hours a day for 1 week for patients with MH and 2 weeks for patients with MHRD.

Pathological exam of the 2 types of commercial hAM graft products was performed to find out the differences in their structures at cellular level.

The statistical analysis was performed using SPSS software (version 22.0; IBM, Armonk, NY). Descriptive statistics were used to obtain mean and standard deviation. Univariate analyses were conducted using Pearson's chi-square test or the Mann–Whitney *U* test. *p* < 0.05 was considered to indicate statistical significance.

## 3. Results

**Figure 1:**
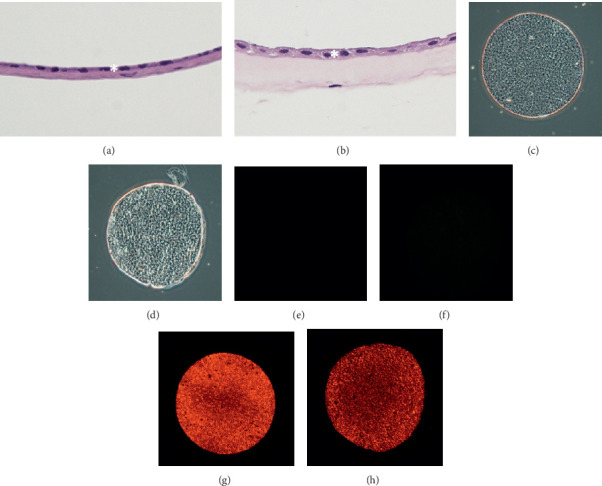
Pathology of dehydrated hAM (a) and cryopreserved hAM (b) with hematoxylin and eosin stain (400x). Note the monolayer epithelium in both hAM (asterisk). Dehydrated hAM is much thinner than cryopreserved hAM. The epithelial cells of dehydrated hAM (c, e, g) and cryopreserved hAM (d, f, h) under the microscope. The epithelial cells were confirmed devitalized by staining with calcein AM (e, f), which is retained within live cells and ethidium homodimer-1 (g, h), which enters cells with damaged plasma membrane and undergoes a 40-fold enhancement of fluorescence upon binding to nucleic acids in devitalized cells.

In the pathological exam, both types of hAM graft have a single layer of devitalized epithelial cells (Figures 1(a) and 1(b)). Cryopreserved grafts have thicker stroma than dehydrated hAM grafts. No viable cells were detected in either type of hAM graft by staining with calcein AM and ethidium homodimer-1 dye (Figures 1(c)–1(h)).

**Figure 2:**
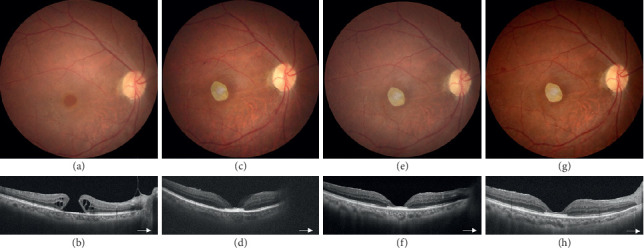
Preoperative fundus photograph (a) and optical coherence tomography (OCT) image (b) of a primary macular hole (MH) in a 69-year-old male. The size of the MH was about 860 *μ*m. Macular hole sealed after transplantation of a dehydrated human amniotic membrane (hAM) graft. One-month postoperative fundus photograph and OCT (c, d), 3-month postoperative fundus photograph and OCT (e, f), and 6-month postoperative fundus photograph and OCT (g, h) showed the hAM graft in place with retinal tissue grew on top of the graft.

**Figure 3:**
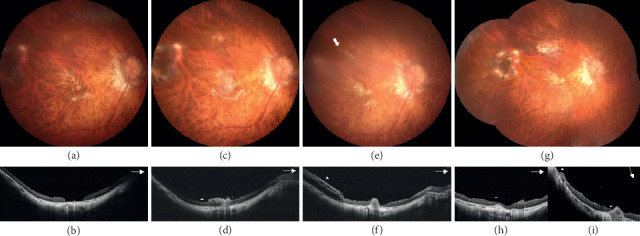
Preoperative fundus photograph (a) and optical coherence tomography (OCT) image (b) of a persistent macular hole (MH) for 26 years in a 59-year-old female. She had received vitrectomy with membrane peeling 2 times for macular retinal detachment before. The size of the MH was about 1250 *μ*m. Macular hole sealed after transplantation of a cryopreserved human amniotic membrane (hAM) graft. One-month postoperative fundus photograph (c) and OCT (d) showed the hAM graft in place connecting 2 ends of MH with partial hAM at retina surface. An extramacular hole (e) (arrow) with retinal detachment (f) (arrowhead) developed 2 months after the surgery. The extramacular hole was sealed, and retina re-attached with dehydrated hAM transplantation in the second surgery in oil phase (g)–(i). OCT (i) showed the extramacular hole was sealed with the hAM graft adhered to retinal pigment epithelium (arrowhead).

**Figure 4:**
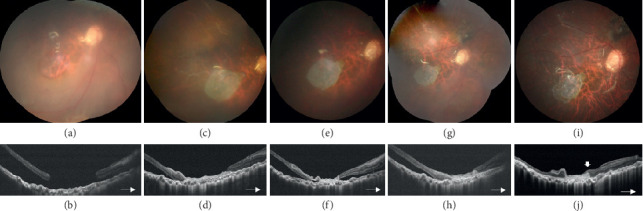
Preoperative fundus photograph (a) and optical coherence tomography (OCT) image (b) of a recurrent macular hole retinal detachment (MHRD) in a 41-year-old male who had received scleral buckle, vitrectomy, and silicone oil tamponade for traumatic macular hole and retinal detachment. The size of the MH was about 3285 *μ*m. Macular hole sealed, and retina was attached after transplantation of a cryopreserved human amniotic membrane (hAM) graft. Two-week postoperative fundus photograph and OCT (c, d), 1-month postoperative fundus photograph and OCT (e, f), 3-month postoperative fundus photograph and OCT (g, h), and 6-month postoperative fundus photograph and OCT (i, j) showed the hAM graft in place with tight adherence to retinal pigment epithelium, and retinal tissue grew on top of the graft (arrow). Subretinal fluid subsided gradually.

In total, 17 patients, 9 males and 8 females, were included in this study. The demographic data and characteristics of all patients were collected (Table 1). Their mean age was 62.1 ± 10.0 years (range 41–81 years). Fourteen patients (82.4%) had MH, and 3 patients (17.6%) had MHRD. Among the 14 patients with MH, 11 patients had persistent MH and 3 had primary MH (Figure 2). Among the patients with MHRD, two were recurrent after previous vitrectomy and one was primary. Among the 17 patients, 9 (52.9%) had a history of MHRD. Thirteen patients (77.5%) had received one to three times of vitrectomy in the past. The average axial length of the affected eye was 27.9 ± 3.4 mm (range 22.30–32.57 mm). Eleven cases (64.7%) were highly myopic, with axial lengths ranging from 27.80 to 32.57 mm. The mean preoperative BCVA was 1.32 ± 0.57 logMAR (range 0.7–3 logMAR, 6/30 to hand motion by the Snellen chart). The mean MH size was 1072.1 ± 666.0 *μ*m (range 230–3285 *μ*m). Cryopreserved hAM grafts were used in 10 patients, and dehydrated grafts were used in 8 patients (one patient received surgery twice with a cryopreserved hAM graft for MH and a dehydrated hAM for extramacular hole, Figure 3). Fourteen patients had 20% SF_6_ tamponade. Two of the patients with recurrent MHRD had silicone oil tamponade (Figure 4), and one patient with persistent MH had the hAM graft inserted under the oil phase.

After following up patients for at least 6 months, 13 cases (76.5%) including 3 cases of MHRD had sealed MHs. There was no significant difference in the MH closure rate between patients with cryopreserved and dehydrated hAM grafts (80.0% vs. 71.4%, *p*=0.68) (Table 2). Among the patients with sealed MHs, 1 patient had partial MH closure with an elevated hAM graft at the nasal edge of the hole after the first surgery and received operation again 1 month later to reposition the old hAM graft and implant a new graft to cover the temporal edge of the hole. The MH was sealed after the second surgery. Another patient had MH closure after the first hAM graft transplantation, but an extramacular hole over the superior arcade with subretinal fluid developed 1 month later. A second hAM graft transplantation to the extramacular hole under the oil phase was performed, and the retina was reattached (Figure 3).

**Figure 5:**
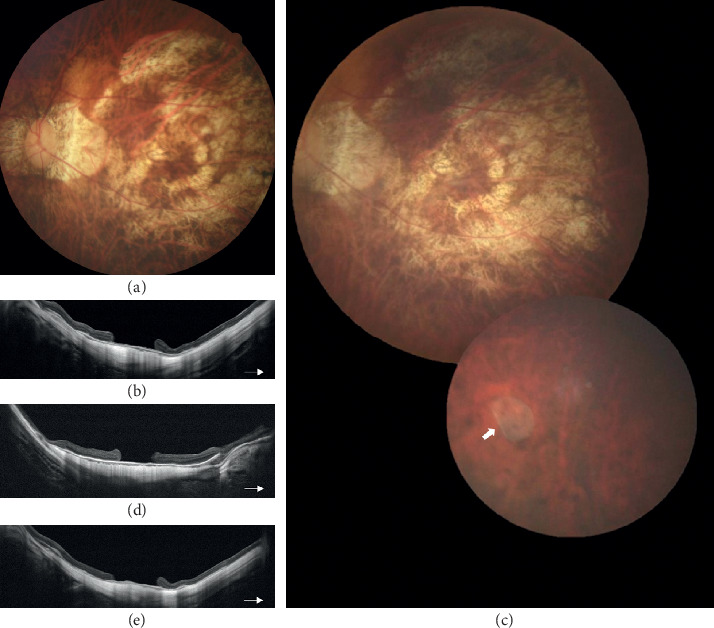
Preoperative fundus photograph (a) and optical coherence tomography (OCT) image (b) of a persistent macular hole (MH) in a 65-year-old male with previous vitrectomy for macular hole retinal detachment (MHRD) 13 years ago. The size of the MH was about 1530 *μ*m. Dislocation of the dehydrated hAM graft inferiorly was noted after the surgery (c) (arrow). Persistent MH was noted in the 1-month and 5-month follow-up OCT (d, e).

The average BCVA of cases with sealed MHs improved from 1.38 ± 0.62 to 1.12 ± 0.47 logMAR (*p*=0.03). When comparing the final visual outcome or visual gain of patients receiving cryopreserved or dehydrated hAM grafts, there were no significant differences between the two groups (change in the BCVA of cryopreserved vs. dehydrated hAM in logMAR: 0.19 ± 0.37 vs. 0.15 ± 0.41, *p*=0.85). In the other 4 cases of persistent MH, 3 cases (16.7%) had hAM graft dislocation (Figure 5), and 1 case (5.6%) had a reopened MH with hAM graft contraction to the nasal and inferior edge of the MH. BCVA remained stable (1.13 ± 0.47 to 1.22 ± 0.39 logMAR, *p*=0.27) in these patients. There was no significant difference in visual gain between highly myopic (axial length ≥26.5 mm) and nonhighly myopic (axial length <26.5 mm) patients (change in BCVA in logMAR: 0.11 ± 0.35 vs. 0.20 ± 0.41, *p*=0.61). No significant difference in visual gain was found between patients with large MHs (≥1000 *μ*m) or small MHs (<1000 *μ*m) (change in BCVA in logMAR: 0.17 ± 0.40 vs. 0.12 ± 0.35, *p*=0.82). After excluding those with MHRD and silicone oil tamponade, MH sizes were significantly larger in patients with graft dislocation, reopened MH, and partial closure (*n* = 5, 1212.6 ± 290.7 *μ*m) than in those with sealed MHs (*n* = 8, 844.8 ± 245.6 *μ*m, *p*=0.03), while age and axial length were not significantly different between the 2 groups.

None of these eyes showed signs of infection, inflammation, or rejection during the follow-up period. No marked regression in either graft type was noted in this short-term follow-up.

## 4. Discussion

In this short case series, we found no significant differences in the anatomical or functional outcomes between the two types of hAM graft. Human amniotic membrane is a fetal tissue composed of epithelium, stroma, and a thick basement membrane. Studies demonstrated that hAM secretes growth factors and adhesion molecules, which facilitate conjunctival and corneal re-epithelialization [28]. Processed hAM has been approved by the United States Food and Drug Administration for ocular surface reconstruction as a kind of allograft and is extensively applied to ocular surface diseases such as pterygium or corneal ulcers [14]. Compared to hAM allografts from a tissue bank, which have uneven surfaces and irregular shapes and are not always readily available, commercial hAM grafts have a flat surface, are perfectly round or rectangular with known sizes, are easier to manipulate, and are readily available in the operating room. Although there were no significant differences in functional or anatomical outcomes between the 2 grafts, differences do exist in handling and storage of the grafts. Dehydrated hAMs are stored at room temperature, which make them easier to transport and preserve. They need to be rehydrated with a small droplet of balanced salt solution before further manipulation. Cryopreserved hAMs must be stored in the frozen state and are expensive in terms of transportation. Cryopreserved grafts are attached to carrier paper, which make them easier to punch and can be directly cut into an optimal size without additional rehydration. During implantation, the stromal side of the cryopreserved graft tends to adhere better to RPE and is spongier, allowing for some fine compression.

In our case series, the average rate of anatomical MH closure after surgery was 76.5% (71.4% in MH and 100% in MHRD) for both types of grafts. In these cases, sealed MHs could be observed at the first follow-up (2 weeks postoperatively). Although cryopreserved hAMs have better preserved structural and biochemical integrity than dehydrated hAMs, we found no significant difference in healing rates or in the regeneration of the outer retina. These grafts acted as scaffolds for glial cell proliferation above the graft as in case 14 (Figure 2) and also fillers to approximate the retina around the hole in all sealed cases. However, layers of external limiting membrane, ellipsoid zones, and interdigitation zones were still disorganized or did not regrow as observed by OCT. The improvement in BCVA, as observed in patients with internal limiting membrane free grafts or free retina grafts with no outer retinal restoration, may be due to the improved sensitivity and decreased scotoma when the halo of the elevated retina around the hole was flattened and approximated [29, 30]. Future functional tests with microperimetry in patients receiving hAM transplantation for MH might be helpful in detecting changes in retinal function around the rim of MHs.

In this short-term study, graft contracture with parafoveal atrophy was found around the hole in four highly myopic cases (average axial length 29.73 ± 2.33 mm, range 27.80–32.57 mm) in the first month after surgery. We hypothesized that the potential contributing factors may include preexisting myopic maculopathy, graft insertion-related surgical trauma, and hAM graft contracture. To minimize the surgical trauma to the RPE, we slid the graft into the subretinal space as gently as possible and avoided excessive manipulation in all cases. Parafoveal atrophy did not develop in the other 3 cases with graft dislocation, 2 of which were highly myopic. Thus, we do not think that surgical trauma itself can fully explain this phenomenon. We hypothesized that the strong adhesion of the stromal side of the hAM graft to RPE may drag the pigment and cause parafoveal atrophy during graft contracture. The mechanism of atrophy in these cases still needs further investigation (*British Journal of Ophthalmology*, submitted).

Unlike the hAM graft for ocular surface reconstruction that dissolves within 2 months and disintegrates by matrix metalloproteinase produced by migrating epithelial cells [31, 32], total absorption of hAM grafts was not observed in our cases in the follow-up period from 6 to 13 months. We initially used hAM grafts in 4 cases with primary MHs because we thought that the graft tissue would be nearly disintegrated after transplantation, like in the cases reported by others [18]. However, the graft shrank only a little in all of our cases, and therefore, we stopped using hAM grafts for primary MHs. We postulated that lack of certain inflammatory cytokines or enzymes, as well as lack of frequent mechanical rubbing of eyelids (as is seen in corneal surgery) probably results in a longer time for graft regression. The long-term fates of the graft and MH need further studies.

Three cases presented with graft dislocation after surgery in our study (cases 5, 6, and 13). The cryopreserved grafts were used for two cases, and the dehydrated graft was used for one case; all graft dislocation was noted on the first postoperative day. All 3 dislocated cases had grafts inserted 360° underneath the retina and used SF_6_ as the final modality of fluid-gas exchange. Compared to those without dislocation, the only factor for these graft-dislocated eyes was the larger size of the hole. Grewal et al. suggested the use of a flap 0.5-disc larger than the hole and use of liquid perfluorocarbon to stabilize the autologous retinal flap routinely and avoid graft dislocation [13]. Cutting the graft into an optimal and precise size can be very challenging, especially for large MHs. In our series, 4 cases (cases 3, 4, 8, and 17) with partial grafts positioned above the edge of the MH still sealed well. In our experience, leaving part of the graft above the retina does not necessarily lead to anatomical failure.

Our study was limited by the small sample size. Further studies with longer follow-up periods are needed to confirm the clinical application of hAM grafts and evaluate the differences between the two types of hAM graft.

## 5. Conclusion

This preliminary case series showed that both dehydrated and cryopreserved hAM grafts were feasible alternatives for MH and MHRD. Both approaches have similar anatomical and functional outcomes. The surgical procedure provides a high sealing rate for persistent MH, MHRD, and giant MHs. However, grafts may dislodge when the size of the MH is large.

## Figures and Tables

**Table 1:** Clinical and demographic information of 17 patients.

Patient	Age (y/o)	Sex	Affected eye	Graft type	Diagnosis	MH size (um)	Time (yr) for MH	Previous VT (diagnosis)	Axial length (mm)	Pre-OP BCVA	Post-OP BCVA	Follow-up length (mo)	Tamponade material	Post-OP findings
1	81	M	OS	Cryopreserved	Persistent MH	924	12	+(MH)	24.52	2/60	2/60	11	20% SF6	Sealed MH
2	71	M	OS	Cryopreserved	Persistent MH	902	7	+(MHRD)	24.33	6/60	6/60	8	20% SF6	Sealed MH
3	67	M	OD	Cryopreserved	Persistent MH	1072	5	+(MHRD)	32.57	5/60	3/60	7	20% SF6	Sealed MH with PFA
4	66	M	OD	Cryopreserved	Persistent MH	1195	7	+(MHRD)	28.27	6/30	6/30	6	20% SF6	Sealed MH
5	60	F	OD	Cryopreserved	Persistent MH	1500	8	+(MHRD)	30.13	6/60	6/60	6	20% SF6	Persistent MH, graft dislocation
6	62	F	OD	Cryopreserved	Persistent MH	975	8	+(MH)	25.02	6/60	5/60	6	20% SF6	Persistent MH, graft dislocation
7	55	M	OD	Cryopreserved	Primary MH	550	0.1	—	30.71	3/60	6/30	10	20% SF6	Sealed MH with PFA
8	41	M	OD	Cryopreserved	Recurrent MHRD	3285	1.5	+(Traumatic MHRD)	23.34	HM	CF	13	SiO	Sealed MH with attached retina
9	55	F	OS	Cryopreserved	Primary MHRD	640	33	—	28.17	6/60	6/30	8	20% SF6	Sealed MH with attached retina
10	69	F	OD	Dehydrated	Persistent MH	905	16	+(MHRD)	31.50	6/30	6/60	13	20% SF6	Reopen MH
11^*∗*^	65	F	OS	Dehydrated	Persistent MH (1^st^ OP) Partially-sealed MH (2^nd^ OP)	1153/737 (1^st^/2^nd^ OP)	5	+(MHRD)	27.80	5/60	6/60	10/9 (1^st^/2^nd^ OP)	20% SF6 (1^st^ & 2^nd^ OP)	Sealed MH with PFA
12	70	F	OS	Dehydrated	Persistent MH	459	4.5	+(MHRD)	27.86	CF	CF	9	20% SF6	Sealed MH with PFA
13	65	M	OS	Dehydrated	Persistent MH	1530	12	+(MHRD)	31.02	1/60	1/60	6	20% SF6	Persistent MH, graft dislocation
14	69	M	OD	Dehydrated	Primary MH	860	0.5	—	22.30	3/60	3/60	8	20% SF6	Sealed MH
15	57	F	OD	Dehydrated	Primary MH	796	2	—	23.78	6/60	6/30	6	20% SF6	Sealed MH
16	43	M	OS	Dehydrated	Recurrent MHRD	230	1.5	+(MHRD)	31.24	CF	6/60	8	SiO	Sealed MH with attached retina
17^*∗∗*^	59	F	OD	Cryopreserved (1^st^) Dehydrated (2^nd^)	Persistent MH (1^st^ OP) Extramacular hole RD (2^nd^ OP)	1250/1500 (1^st^/2^nd^ OP	26	+(MHRD)	31.23	6/60	6/30	7/6 (1^st^/2^nd^ OP)	SiO (1^st^ and 2^nd^ OP)	Sealed MH (1^st^) Sealed extramacular hole with attached retina (2^nd^)

^*∗*^This patient had received 2 times of hAM graft transplantation. Persistent macular hole with elevated hAM graft at the margin of MH was noted after the 1^st^ surgery. Sealed MH was noted finally after the 2^nd^ surgery. ^*∗∗*^This patient had received 2 times of hAM graft transplantation. Extramacular hole with retinal detachment was noted after the 2^nd^ surgery. Sealed macular hole and extramacular hole with attached retina was noted after the 2^nd^ surgery. y/o: year-old; M: male; F: female; MH: macular hole; MHRD: macular hole retinal detachment; OP: operation; RD: retinal detachment; yr: year(s); VT: vitrectomy; BCVA: best-corrective visual acuity; HM: hand movement; CF: counting finger; LP: light perception; mo: month(s); SF6: sulfur hexafluoride; SiO: silicone oil; PFA: parafoveal atrophy; hAM: human amniotic membrane.

**Table 2:** Comparison between clinical data of two types of human amniotic membrane graft.

Type of hAM	Cryopreserved	Dehydrated	*p* value
Number of cases^*∗*^	10	7	
Sex (M : F)	6 : 4	3 : 4	
Age (y/o)	61.70 ± 10.72	62.57 ± 9.69	0.87
Axial length (mm)	27.83 ± 3.32	27.93 ± 3.70	0.95
Size of MH (*μ*m)	1229.30 ± 774.54	847.57 ± 427.69	0.26
Sealed MH (%)	8 (80%)	5 (71%)	0.68
Visual gain (logMAR)	0.19 ± 0.37	0.15 ± 0.41	0.85

^*∗*^One patient received cryopreserved hAM graft for MH, and dehydrated hAM graft for extramacular hole was enrolled in the cryopreserved group. M: male; F: female; y/o: year-old; MH: macular hole.

## Data Availability

The data used to support the findings of this study are available from the corresponding author upon request.
